# Ensuring young voices are heard in core outcome set development: international workshops with 70 children and young people

**DOI:** 10.1186/s40900-020-00202-9

**Published:** 2020-05-06

**Authors:** Frances C. Sherratt, Heather Bagley, Simon R. Stones, Jenny Preston, Nigel J. Hall, Sarah L. Gorst, Bridget Young

**Affiliations:** 1 Department of Health Services Research, Institute of Population Health Sciences, University of Liverpool, Room 223, Second Floor, Block B, Waterhouse Building, 1-5 Dover Street, Liverpool, L69 3GL UK; 2 Institute of Translational Medicine, University of Liverpool, Liverpool, UK; 3 School of Healthcare, University of Leeds, Leeds, UK; 4 NIHR Alder Hey Clinical Research Facility, Alder Hey Children’s NHS Foundation Trust, Liverpool, UK; 5 University Surgery Unit, Faculty of Medicine, University of Southampton, Southampton, UK

**Keywords:** Children, Young people, Core outcome set, Outcomes, Patient and public involvement

## Abstract

**Plain English summary:**

Researchers test treatments to ensure these work and are safe. They do this by studying the effects that treatments have on patients by measuring outcomes, such as pain and quality of life. Often research teams measure different outcomes even though each team is studying the same condition. This makes it hard to compare the findings from different studies and it can reduce the accuracy of the treatment advice available to patients. Increasingly, researchers are tackling this problem by developing ‘core outcome sets’. These are lists of outcomes that all researchers working on a given condition should measure in their studies. It is important that patients have a voice in the development of core outcome sets and children and young people are no exception. But their voices have rarely been heard when core outcome sets are developed. Researchers are trying to address this problem and make sure that core outcome sets are developed in ways that are suitable for children and young people. As a first step, we held two international workshops with children and young people to listen to their views. They emphasised the importance of motivating young people to participate in developing core outcome sets, making them feel valued, and making the development process more interactive, enjoyable and convenient. We hope this commentary will encourage researchers to include children and young people when developing core outcome sets and to adapt their methods so these are suitable for young participants. Future research is important to examine whether these adaptations are effective.

**Abstract:**

**Background**

Different research teams looking at treatments for the same condition often select and measure inconsistent treatment outcomes. This makes it difficult to synthesise the results of different studies, leads to selective outcome reporting and impairs the quality of evidence about treatments. ‘Core outcome sets’ (COS) can help to address these problems. A COS is an agreed, minimum list of outcomes that researchers are encouraged to consistently measure and report in their studies. Including children and young people (CYP) as participants in the development of COS for paediatric conditions ensures that clinically meaningful outcomes are measured and reported. However, few published COS have included CYP as participants. COS developers have described difficulties in recruiting and retaining CYP and there is a lack of guidance on optimising COS methods for them. We aimed to explore CYP’s views on the methods used to develop COS and identify ways to optimise these methods.

**Main body**

This commentary summarises discussions during two workshops with approximately 70 CYP (aged 10–18 years old) at the International Children’s Advisory Network Research and Advocacy Summit, 2018. Delegates described what might motivate them to participate in a COS study, including feeling valued, understanding the need for COS and the importance of input from CYP in their development, and financial and other incentives (e.g. certificates of participation). For Delphi surveys, delegates suggested that lists of outcomes should be as brief as possible, and that scoring and feedback methods should be simplified. For consensus meetings, delegates advised preparing CYP in advance, supporting them during meetings (e.g. via mentors) and favoured arrangements whereby CYP could meet separately from parents and other stakeholders. Overall, they wanted COS methods that were convenient, enjoyable and engaging.

**Conclusion**

This commentary points to the limitations of the methods currently used to develop COS with CYP. It also points to ways to motivate CYP to participate in COS studies and to enhancements of methods to make participation more engaging for CYP. Pending much needed research on COS methods for CYP, the perspectives offered in the workshops should help teams developing COS in paediatrics and child health.

## Background

### What is an outcome and what is a core outcome set?

An outcome of treatment is defined as “*a measurement or observation used to capture and assess the effect of treatment, such as assessment of side effects (risks) or effectiveness (benefits)*” [1]. Gaps in knowledge about which treatment outcomes are most important to children, young people, parents/carers and health professionals, can result in studies measuring outcomes that are of little relevance from their perspective. Ultimately, this can result in evidence that is of limited use for informing treatment decisions for children and young people (CYP). Table 1 provides a list of abbreviations used in this article.

**Table 1:** List of abbreviations

COMET	Core Outcome Measures in Effectiveness Trials
COS	Core outcome set(s)
CYP	Children and young people
iCAN	International Children’s Advisory Network
NHS	National Health Service
NIHR	National Institute for Health Research
PoPPIE	People and Patient Participation, Involvement and Engagement
PPI	Patient and public involvement
YPAG	youth patient advisory group

Studies of the same condition often measure inconsistent outcomes, making it hard to synthesise the results across studies (i.e. in meta-analysis or systematic reviews) (e.g. [2]). Such inconsistency in outcome measurement also fuels selective outcome reporting (i.e. cherry picking of findings), whereby researchers select which outcomes to publish depending on the statistical significance or the magnitude of an effect [3]. Ultimately, selective outcome reporting impairs the quality of evidence, and leads to distrust in research and research waste [4–7]. Moreover, as noted above, the outcomes measured in studies need to be relevant and meaningful to key stakeholders (such as patients, parents or carers, health professionals, researchers etc) if the reported results are to translate into evidence that can improve decisions about treatment and care [8].

Developing and applying an agreed set of outcomes, known as a core outcome set (COS), can help to address the above problems [9, 10]. A COS represents the minimum outcomes that should be reported in studies in a given clinical area, or on a given population; researchers are not restricted only to the outcomes in a COS and can measure and report additional outcomes. Table 2 shows an example of a COS developed for uncomplicated acute appendicitis research in CYP [11].

**Table 2:** An example core outcome set (COS) [11]

The minimum outcomes to measure and report for studies investigating treatment of uncomplicated acute appendicitis in children and young people [11]	
• Bowel obstruction	
• Wound infection	
• Wound complication	
• Negative appendicectomy	
• Recurrent appendicitis	
• Intra-abdominal abscess	
• Antibiotic failure	
• Child’s quality of life	
• Patient stress / psychological distress	
• Time away from full activity	
• Length of hospital stay	
• Readmission to hospital	
• Reoperation (including interventional radiology procedure)	
• Death	

### How is a core outcome set developed?

An increasing number of COS are being developed, and methodological research and recommendations to optimise COS development are available [1]. These recognise the value of including patients in the development of COS, alongside other stakeholders, to ensure that COS are relevant to patients. Various methods have been used to reach agreement, or ‘consensus’, among stakeholders about which outcomes that should be measured and reported for a given condition. These include: semi-structured group discussions, unstructured group discussions, consensus development conferences, systematic reviews, Delphi techniques, surveys, nominal group techniques, interviews, and combinations of such methods [12]. Delphi surveys with consensus meetings are the most common combination of COS development methods [13]. These involve two or more survey rounds with the aim of reaching consensus among stakeholders on which outcomes are the most important to include in a COS [1]. Participants are typically also provided with the opportunity to propose new outcomes, if they feel that an outcome of importance is not already included in the initial long list of outcomes presented in the round one Delphi survey. Delphi surveys enable large numbers of geographically disperse participants to take part [1] and offer anonymity, which can minimise the influence of power differentials between stakeholder groups [14]. Consensus meetings commonly follow Delphi surveys to enable participants to discuss the results of the Delphi survey and undertake further voting if needed, sometimes using nominal group techniques [15] to agree on the final core outcome set [1].

In discussing the process of COS development a distinction is often drawn between decisions about ‘what’ outcomes should go in a COS, and decisions about ‘how’ to measure those included outcomes (i.e. which instruments or tools are used to measure outcomes such as pain, quality of life etc., and the time intervals at which the measures are taken). Deciding what outcomes should go in a COS tends to be the first step in the development process and so that is our focus here.

### Current challenges in developing core outcome sets with children and young people

Various methods have been used to develop a COS, but it is unclear which methods are most suitable and effective when developing COS for paediatric conditions. Adult participants have been increasingly included in studies to develop COS [1]. In contrast, of 63 published COS studies relevant to paediatric conditions in the COMET database up to October 2019 (excluding COS focussed on infants), only 8 (13%) have had direct input from CYP. Rather than including CYP directly, many study teams have only included parents or carers and health professionals to speak on behalf of CYP. Several COS developers who have tried to include CYP directly have described challenges in recruiting and retaining them as participants [11, 16, 17], with the extent of their input varying widely between studies, and sometimes tailing off markedly during the later stages of the development process [11, 16–19]. Methods used in these studies such as Delphi surveys were initially designed for adults, raising questions about whether these are suitable and effective for use with CYP. It is essential to optimise CYP’s input in COS development to ensure that outcomes of importance to them are not overlooked.

Throughout this article we use the abbreviation CYP rather than referring to ‘patients’. This is to recognise that not all COS are developed for patients, and that some COS are intended for children/young people for whom the term ‘patient’ is not appropriate (e.g. disabled children and young people) or contested (e.g. children and young people who use mental health services). Other COS are developed to assess interventions or services that are implemented in community or social care settings where, similarly, the term ‘patient’ is not appropriate (e.g. services aiming to improve outcomes for children affected by domestic abuse) [20].

### Aims

As most COS intended for use with CYP have had little direct input from CYP themselves, further research is needed to identify optimal methods to recruit and retain CYP in COS development. Pending such research becoming available, we consulted with CYP via workshops at the International Children’s Advisory Network (iCAN) Research and Advocacy Summit held in 2018. We aimed to establish their views on consensus methods currently used to develop COS such as Delphi surveys and consensus meetings. Our goal was to provide COS developers with pointers on recruiting and retaining CYP in COS studies for paediatric conditions.

## Main text

We held two workshops entitled ‘Choosing meaningful outcomes for research: making sure young people have their say’ at the international iCAN Summit in Edinburgh, UK, on 12th July 2018. iCAN is an international consortium of youth advisory groups established in 2015. The consortium aims to provide a way for the perspectives of young patients to inform the decision-making processes of regulatory agencies and pharmaceutical companies in the development of clinical trials and treatments, and to facilitate researchers and clinicians in consulting with CYP when designing studies [21]. iCAN comprises 21 youth patient advisory groups (YPAGs) with members aged 8–25 who are patients at local research institutions and hospitals or have links to these. To our knowledge, iCAN is the only international CYP organisation with a health-related focus. Any person who is a member of a YPAG that is part of the consortium can apply for a scholarship to attend an iCAN Summit. Most YPAGs within iCAN are based in North America and Europe, although there are some groups in Africa. More information about iCAN is available on their website [22] and in a brochure on the 2018 Edinburgh iCAN Research & Advocacy Summit [23].

### Who participated in the workshops?

The week-long Edinburgh summit was hosted by the National Heath Service Resarch Scotland - Children’s Research Network in partnership with GenerationR-NIHR (National Institute for Health Research) England. Our COS workshops were open to all CYP summit delegates. Each delegate received a brief description in advance of the workshops, which explained that those attending the workshops would learn about outcomes in research, some of the problems with outcomes in research and potential solutions to these problems. It also explained that delegates would have the opportunity to help to shape how CYP are involved in agreeing meaningful outcomes in future research. As the workshops were a consultation activity rather than a research activity, we did not take a formal register of delegages or collect data on their demographic characteristics. However, a ‘head count’ indicated that approximately 70 delegates attended the workshops. The iCAN organisers informed us that delegates were aged between 10 and 18 years, were mostly from high income socio-economic backgrounds within countries across Europe and North America and included healthy CYP and those with experience of acute and chronic conditions. The iCAN organisers also informed us that delegates were often motivated to join CYP advisory groups and attend the summit because they had an interest in undertaking health-related educational courses and careers, although others attended simply to share their voice and experience. While parents attended the summit with their child, they did not take part in the COS workshops as our focus was on the views of CYP.

### How were the workshops organised and facilitated?

Each of the two workshops followed the same session plan and lasted 30–40 minutes. The workshop discussions focussed on research methodology rather than on delegates’ personal experiences of illness or treatment. As research methodology is rather an abstract topic, it was unlikely that any delegates would experience distress afterwards. However, iCAN conference organisers experienced in working with CYP were available to support them if needed.

BY and HB gave a short presentation at the beginning of each workshop. This had been developed by BY, HB and FS and explained what an outcome is, what a COS is, what methods are used to develop COS, and the ways that CYP currently provide input into COS studies. Delegates also viewed a brief animated video produced by The COMET Initative, that summarised, in plain English, why COS are needed and how they are developed [24].

**Fig. 1:**
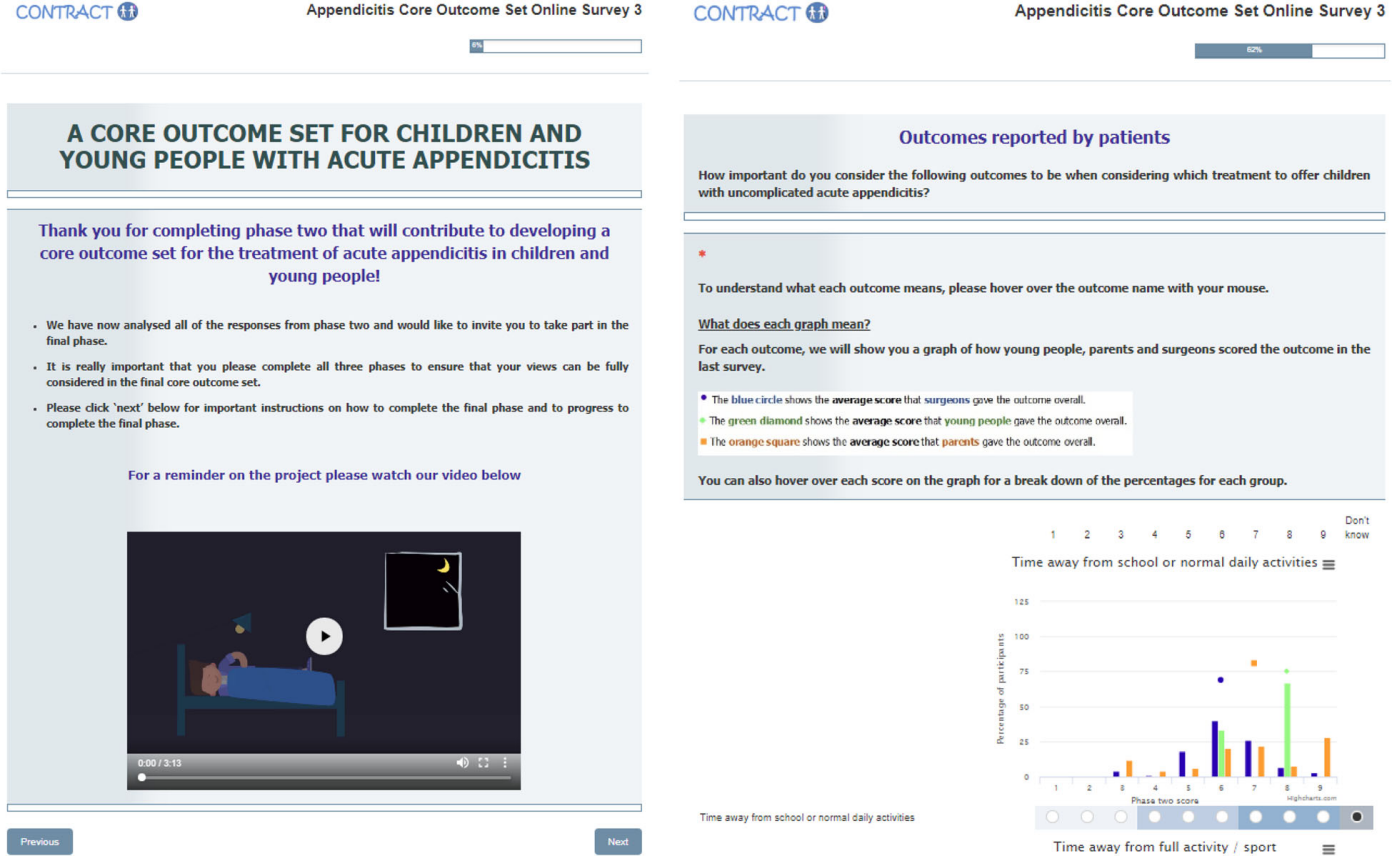
Screenshots from a Delphi survey website used to develop a core outcome set

We worked in small groups of approximately 6–8 delegates within each workshop. Half of the groups (the ‘blue groups’) were provided with screenshots of the guidance to Delphi survey participants and feedback to participants (Fig. 1) that had been used with CYP in a previous COS [25]. The survey software used in this example was developed by The National Perinatal Epidemiology Unit (University of Oxford) and was similar to Delphi survey software developed by other providers. We asked delegates in the ‘blue groups’ to discuss: (i) What would you think if you were asked to complete a survey like this? (ii) How could the survey be improved? (iii) Are there better ways of collecting the views of children and young people on outcomes of importance. The other groups (the ‘green groups’) were asked to consider how consensus meetings could be improved. In particular, we asked them to discuss: (i) What would you think if you were aksed to take part in a consensus meeting? (ii) How could the meeting format be improved? (iii) Are there better ways of collecting the views of children and young people on outcomes of importance. Each group had at least one facilitator (BY, HB, FS, SS, or JP) to prompt discussion. All facilitators were professional contacts of BY or HB and all had experience of leading discussions with CYP in similar contexts, such as patient and public involvement (PPI) activities, or COS development. All groups were asked to discuss their respective topics for 20 min. We used prompts (summarised in Table 3) to facilitate the discussions. Each group was provided with a paper table cloth and marker pens for writing comments/doodles, and emoji stickers to flag important discussion points; facilitators also made notes during the discussions.

**Table 3:** Overview of prompts used to facilitate workshops

**1. Both Delphi survey and consensus meeting group prompts**	
• What would you think if you were asked to take part?	
• What might gain your interest in taking part in a core outcome set study?	
• How easy would you find it to talk about outcomes?	
• Are there better ways of collecting the views of children and young people on outcomes of importance?	
• How could the method (Delphi survey or consensus meeting) be improved?	
• What specific considerations should we be aware of for children and young people?	
**2. Delphi survey – specific discussion group prompts**	
• What information would be helpful?	
• What do you think about how the information is presented? Website, scoring, feedback.	
• What would you think about having to consider the outcomes and score them?	
• What would you think about voting on an outcome you may not have had experience of?	
• What do you think about having to complete the survey two or three times? What would keep you engaged between surveys?	
• What do you think about considering the feedback on votes from other stakeholders?	
• How would you feel about rescoring? How would you feel about being asked why you had rescored?	
**3. Consensus meeting – specific discussion group prompts**	
• What do you think happens at a meeting and what would you like to happen?	
• What would encourage or discourage you to go? Worries/concerns?	
• What would help you to prepare for the meeting? Pre-meeting useful?	
• Format of meeting – Who should be there (i.e. stakeholder groups)? Pros and cons of options. Where should people sit (e.g. mixed or separate groups of children, young people and parents)?	
• What do you think about the voting system? Suggestions?	
• What do you think about considering the feedback on votes from other stakeholders? How would you feel about rescoring?	
• What might reassure you that your opinion was really important at that meeting?	

Following the workshops, FS drafted a document to capture the comments that delegates in all groups wrote on the table cloths, and to summarise the group discussions. FS circulated this document to the other facilitators, who each added further notes on the comments and discussions from their groups. FS finalised the document, which ultimately described delegates’ views on Delphi surveys and consensus meetings. We summarise these views in the subsequent sections.

### Motivating children to participate in COS development

In both workshops, delegates demonstrated excellent understanding of the workshop objectives and seemed enthusastic about including CYP in COS development. Delegates discussed factors that might motivate CYP to participate in COS development. Suggestions were similar, regardless of whether groups had discussed the use of Delphi surveys or consensus meetings. Many delegates described that being made to feel valued and knowing they are making a useful contribution would be key in motivating CYP to participate. They wanted COS developers to communicate how the participation of CYP may contribute to improving their own health and/or the health of others. Specifically, they thought CYP would want to understand why COS are needed, why CYP input in the development of COS is important, and the role of COS in improving treatments. They felt that the COMET Initiative animated video (www.comet-initiative.org/resources/PlainLanguageSummary) provided a clear explanation of COS. They added that such animations were better for CYP than written participant information, and might further encourage CYP to participate. For Delphi surveys, some delegates suggested that including a video on the survey website that created a human interest angle on the need for the COS would help to engage the attention of CYP. For example, one attendee suggested including a video in which a patient could describe their experience of a given condition and how a patient could be affected if ‘the wrong’ outcomes were included in research studies.

Typically, delegates commented that participants should also be offered financial incentives for their time participating in Delphi surveys and consensus meetings. This might include money or pre-paid gift cards. However, a few delegates disagreed, commenting that financial incentives might encourage some CYP to register but not participate, or particpate without really thinking about their responses. One group of delegates suggested such difficulties could be avoided by providing participants with ‘surprise’ gifts over the course of the study, rather than offering financial incentives upfront. One delegate explained that providing CYP with payment for research is not uniformly accepted in all countries and that this should be considered. Several delegates also suggested that a certificate of participation that could be included in their portfolios and mentioned on CVs to enhance their educational and career prospects, would encourage CYP to participate.

For consensus meetings specifically, delegates said that accommodation and travel should either be arranged by the research team, or that families should be offered payment for such costs in advance, rather than waiting to be reimbursed. Delegates also felt that consensus meetings should be well catered for, with meals and refreshments that CYP would enjoy.

### Optimising Delphi surveys for children and young people

#### Delphi survey website format

Delegates suggested that Delphi surveys would likely be suitable for participants aged 12 years and over. They raised questions about the suitability of Delphi surveys for younger participants and commented that this would depend on the individual participant and the content of the survey. They suggested that the Delphi survey software should be accessible on various devices, especially as many said they would complete the survey on their mobile phones. A few delegates also commented that they would prefer an interview or focus group format to completing a survey.

Some felt the example Delphi survey website landing page that we showed was dull and should be more colourful, engaging and visually appealing. They recommended that COS developers obtain input from a Young Persons’ Advisory Group to further develop software and enhance the presentation of Delphi surveys. For example, they advised that participants should be able to place a cursor over questions and hear a spoken description of the outcome and any accompanying explanations. This way participants would have less reading, making the Delphi survey less burdensome to complete. Some delegates suggested gamifying Delphi survey websites. For example, one suggestion was to include options whereby participants could select an avatar and collect virtual tokens as they completed the survey to make the experience more engaging. Several delegates also suggested that support for completing Delphi surveys should be readily avialable to participants via live chat or other means for participants to ask questions.

#### Presenting the long list of outcomes

A key discussion point for delegates was the length of the list of outcomes presented to participants when completing a Delphi survey and how long scoring should take. Unsurprisingly, delegates commented that very long list of outcomes would put them off completing the survey, or lead to them losing interest and scoring outcomes that appeared towards the end of the survey less accurately. They advised limiting surveys to approximately 20 outcomes, or ensuring that the survey should take no longer than around 10–15 min to complete. To make scoring more manageable, one group also suggested presenting each outcome on a separate page, rather than numerous outcomes being presented on the same page.

#### Scoring system

The Grading of Recommendations, Assessment, Development and Evaluations (GRADE) 9-point Likert scale, where outcomes are rated in accordance to their level of importance [26], is one of the most common scoring systems used in the Delphi surveys for COS development [1]. This system was used in the example Delphi survey that we showed to delegates. Most groups felt that a 9-point scale was too complicated and were unclear why it was necessary. They suggested that CYP might find it challenging to score an outcome with so many points and advised simplying the scale, proposing alternative formats, such as a 5-point Likert scale, yes/no responses, and a traffic light system. They also preferred the descriptors used for the GRADE scale (i.e. ‘not very important’, ‘important’ and ‘very important’) to numbers, commenting that the descriptors were more meaningful as an aid to outcome scoring.

#### Feedback between rounds

To move towards consensus among COS study participants, it is important to reconcile differing views. In Delphi surveys, participants are typically provided with feedback in second and subsequent survey rounds, which enables them to consider others’ opinions before re-rating outcomes, and changing their scores if they wish [1]. Delegates felt that it is important to clearly explain the rationale for subsequent survey rounds to participants, and how to complete these. Without this information, delegates believed that participants would be confused about why they were being sent the same survey again and unclear about what was expected of them when rescoring outcomes. In turn, this could discourage participants from remaining in the study. Delegates advised holding subsequent survey rounds promptly after the previous round (e.g. within 1 week). Where gaps were longer they advised distributing study newsletters between survey rounds to maintain participants’ interest in the study.

Delegates also commented on the impact of presenting feedback to CYP on the results from the previous rounds. In Delphi surveys, this entails providing individual participants with their own scores in the previous survey round, and the collated scores of their own stakeholder group (i.e. other CYP) in the previous round, or the collated scores of all stakeholder groups (e.g. CYP, parents and health professionals combined). Some delegates believed that being sent the collated scores may lead younger participants to worry that they have completed the survey incorrectly if the collated scores are different to their own. Or that it could lead participants to conform to how others have scored an outcome, regardless of the importance they personally place on the outcome.

COS developers commonly present feedback on the results from the previous survey rounds as summary statistics (e.g. means or medians), percentages across a Likert scale, graphical distribution of scores (see Fig. 1), or a selection of these formats [1]. The example we showed to delegates included a combination of all three feedback approaches. Although some liked the graphical presentation, many felt that the graphs would be too complicated for children and younger teens to interpret, with one delegate saying it reminded them of doing a maths test. Delegates added that the graphs might discourage CYP from completing the survey. Some asked what the “dots” (the mean scores) were on the graphs and felt these were confusing. Overall, delegates believed that interpreting the graphs would be time consuming and discourage CYP from completing the survey. They favoured simpler approaches such as pie charts or the use of means or percentages only.

### Optimising consensus meetings for children and young people

#### Preparation and support

While several delegates said that simply being invited to participate in a consensus meeting would make them feel valued, others anticipated that the prospect of attending a meeting might lead to CYP feeling intimidated or overwhelmed. They proposed strategies that COS developers could use to prepare and support the participation of CYP. These included presenting participants with a video to introduce the meeting, and offering participants contact with a mentor/advocate who they could speak to before the meeting, and who could also speak on their behalf during the meeting if they did not feel comfortable. Delegates also suggested providing participants with an agenda and time estimations for each part of the meeting so they knew what to expect during the meeting.

#### When and where to hold a meeting

Delegates proposed that consensus meetings should ideally be held at weekends, as it would be difficult to arrange time away from school or college. Some said that a full day meeting would be acceptable if catering was provided at several time points throughout the day, but many believed half day meetings would be preferable. If the travel time to the meeting exceeded two hours some suggested that CYP would be less likely to attend, although providing accommodation (as discussed earlier) might help overcome this.

#### Separate or joint consensus meetings with stakeholder groups

Many delegates ancipated that CYP would prefer meeting separately from other stakeholder groups, as the presence of parents and health professionals might be inhibiting and make CYP feel embarrassed about “saying something wrong”. Delegates suggested that if they were organised into small groups at the meeting with other CYP, they would have opportunity to make friends and feel less inhibited. If a joint meeting with all stakeholder groups was unavoidable, delegates suggested that participants should be placed in groups with a balanced mix of different stakeholders, and that it was important to ensure that the small groups were not dominated by health professionals or parents/carers. Some added that CYP should still be provided with the opportunity to spend time with other CYP during joint meetings.

#### Preferences for discussion and outcome voting

Delegates believed that COS developers should try to make outcome discussion and voting interesting and interactive. They felt that having discussion and voting in small groups, and mixing the discussion of “interesting” and “less interesting” outcomes throughout the meeting might help with this. Several delegates also suggested that the meeting should be broken up with fun activities and games to maintain engagement, whilst adding that fun activities should be clearly demarcated from the more serious activities such as outcome discussion and voting. Delegates added that it was important for participants’ anonymity to be maintained when voting for outcomes.

#### Other consensus meeting considerations

Some delegates proposed alternatives to face-to-face consensus meetings, such as online consensus meeting, to improve accessibility for CYP who might not be able to attend meetings in person. They suggested that live chat could be used to communicate during the meeting and mobile applications (i.e. Android or Apple apps) could enable participants to vote on outcomes remotely.

## Conclusions

Few COS that are relevant for CYP have had direct input from them. Several COS developers who have attempted to include CYP as participants have reported difficulties in recruiting and retaining them [11, 16, 17] and there is currently no guidance on optimal methods for doing so. In both of the workshops that we report on in this commentary, delegates demonstrated an excellent understanding of the workshop objectives and offered valuable insights and recommendations to optimise COS development for CYP. The COMET Initiative established the PoPPIE (People and Public Participation, Involvement and Engagement) Working Group to lead and oversee the participation, involvement and engagement work of the COMET Initiative. Historically, PoPPIE have focused more on adult patient participation in COS development; however, PoPPIE members have an active interest in improving research with CYP and they initiated the idea of holding the workshops described in this commentary. This commentary highlights the important contributions that CYP can make as research advisors in COS studies. Their involvement via the iCAN summit identifies ways to better engage CYP as participants in COS studies and potentially improve the quality of such studies [27].

Perhaps most strikingly, the workshop delegates suggested that CYP would be motivated to participate in COS studies by feeling that they were making a valuable contribution; this seemed more important than financial incentives. They commented that current practices in COS development (e.g. particularly long lists of outcomes in Delphi surveys) may lead to inaccurate responses or discourage CYP from participating or remaining in a study. CYP wanted Delphi surveys to be easily accessible via mobile devices, and for scoring and feedback in second or later rounds to be simplified. They also wanted preparation and support to be offered to CYP participating in consensus meetings and favoured meetings where CYP could discuss outcomes separately from other stakeholders particularly their parents, or at least for meetings to be organised in ways that supported CYP in voicing their perspectives on an equal footing with other stakeholder groups. We nevertheless note that CYP who are less confident or experienced in voicing their perspectives than those in our workshops may wish their parents to be present in discussions.

Based on the perspectives of delegates, we suggest that for meaningful participation of CYP in COS development, current methods needed to be adapted. Otherwise the views of CYP will remain largely unheard and future COS will risk overlooking outcomes that matter to them. Previous work has advocated the importance of methodological work to understand the COS development process from the perspective of patient participants to optimise their engagement and participation [14]. However, the focus to date has been on adult patient participants in COS development. Our work with CYP indicates the distinct challenges of including them as participants and offers some ideas for addressing these challenges.

Methodological research to understand the COS development process from the viewpoint of CYP is particularly needed to identify how best to include them in this process. Such research is important to inform the design of future methods and research is needed to evaluate these methods. Future work should also address how to engage CYP from rarely included groups, such as black and minority ethnic groups and those from socio-economically disadvantaged backgrounds [28–30]. This is especially pertinent as the iCAN delegates we consulted were typically from high income backgrounds and countries. Moreover, some were particularly interested in health-related issues and intended to pursue careers as health professionals. Future research is also needed to produce guidance to help teams optimise participation and involvement of CYP in COS development. Pending such guidance becoming available, we hope this commentary will help COS developers to adapt COS development methods so that these are more suitable for young participants.

## Data Availability

Not applicable.
